# A hospital-based study on complementary and alternative medicine use among diabetes patients in Rajshahi, Bangladesh

**DOI:** 10.1186/s12906-020-03021-3

**Published:** 2020-07-13

**Authors:** Md. Abdur Rafi, Dewan Tasnia Azad, Mridula Bhattacharjee, Nikita Rahman, Kazi Abdul Mubin, Md. Ajijur Rahman, Md. Golam Hossain

**Affiliations:** 1 Rajshahi Medical College, 6000, Rajshahi, Bangladesh; 2 Jashore Medical College, Jashore, 7400 Bangladesh; 3 Kushtia Medical College, Kushtia, 7040 Bangladesh; 4 Department of Pharmacy, University of Rajshahi, -6205, Rajshahi, Bangladesh; 5 Department of Statistics, University of Rajshahi, 6205, Rajshahi, Bangladesh

**Keywords:** Complementary and alternative medicine, Type 2 diabetes mellitus, Hospital-based, Glycemic control, Bangladesh

## Abstract

**Background:**

The use of complementary and alternative medicine (CAM) among type 2 diabetes mellitus (T2DM) patients is increasing worldwide. It can affect optimum glycemic management. This study was to determine the rate and influencing factors of CAM use among diabetes patients as well as their effect on glycemic control.

**Methods:**

This cross-sectional study was conducted among T2DM patients attending the outpatient department of Rajshahi Medical College Hospital. It is a tertiary hospital in the northern part of Bangladesh. A face-to-face interview with a pretested structured questionnaire was used for data collection. Chi-square (χ^2^) test and multivariate logistic regression model were used in this study for data analysis.

**Results:**

Out of 244 T2DM patients, 86 (35.2%) used CAM. Multivariate logistic regression model showed that lower family income group (AOR = 8.7, 95% CI: 2.15–35.22, *p*-value 0.002), having no institutional education (AOR = 3.4, 95% CI: 1.17–9.87, p-value 0.025) and having diabetes for more than five years (AOR = 2.821, 95% CI: 1.34–5.94, p-value 0.006) were the most influential predictors of CAM use. The most commonly used CAMs were herbal products (67.4%) and homeopathic medicine (37.2%). Most of the CAM users (72%) were influenced by friends, neighbors, and family members. The most common reasons behind CAM use were reported to be the belief that CAM helped control diabetes better (44.2%) and easy availability and lower cost (27.9%). More than half of the users reported the efficacy of CAM as ‘nothing significant’, while others reported as somewhat good. 14% of CAM users experienced side-effects, especially gastrointestinal upset. It was observed that using CAM was associated with poor glycemic control (AOR = 2.25, 95% CI: 1.14–4.44, *p*-value 0.018).

**Conclusion:**

Our study demonstrated that some modifiable factors are associated with the use of CAM, and it cannot maintain good glycemic control. So, patients should be made aware of the ineffectiveness and bad effects of CAM by enhancing educational and poverty-alleviating programs.

## Background

Type 2 diabetes mellitus (T2DM) has become a major public health concern worldwide. Over the last two decades, diabetes has substantially increased in terms of an absolute number of years of life lost and years lived with disability [1], and it affects more than 382 million people globally [2]. A large portion of diabetes patients lives in South East Asia [3]. Bangladesh, a developing country of this region, has more than 8.4 million diabetes patients, which constitutes almost 8% of the total adult population of the country [3]. The health system of Bangladesh is inadequate to provide the essential services for this large number of patients.

Recent advances in diabetic care have made good glycemic control possible, but yet modern medications and essential lifestyle modifications cannot cure diabetes rather patients have to continue lifelong therapy. These issues are making patients interested in various complementary and alternative medicines (CAM) [4], which refers to ‘a group of diverse medical and health care systems, practices, and products that are not presently considered to be part of conventional medicine’ [4, 5]. The use of such alternative therapies is increasing in different countries [4, 6–8]. Patients’ demographic characteristics, experience, health beliefs and attitudes, and behavior toward disease and therapies can influence their practice of using CAM therapy [9]. Different categories of CAM were reported to be used by diabetes patients including biological therapies like an herbal and dietary supplement, alternative medical systems like acupuncture or ayurveda, energy therapies like reiki, manipulative and body-based systems like chiropractic or massage, and mind-body interventions like tai-chi or yoga [4, 10].

The effect of CAM use on glycemic control is still controversial. A few evidences are showing that the use of CAM, especially different herbs and supplements can be beneficial for diabetes management and self-care [4, 11]. However, these herbal therapies may have side-effects if not used correctly [12]. Moreover, increasing dependence on alternative therapy may have a negative impact on glycemic control and patients’ quality of life [6]. As diabetes is a self-managed disease, a patient-centered approach for diabetes management is more effective for therapeutic compliance [13]. Their attitude and practice of using alternative therapy may influence medication compliance and glycemic control. Potential physiological impacts like medication interaction with CAM therapies and their side-effects should also be taken into account [11]. Understanding the prevalence and pattern of use of different complementary and alternative therapies and beliefs and attitudes of patients toward these is important for effective diabetes management.

CAM is widely used in Bangladesh both for the prevention and treatment of various diseases. It is estimated that more than two-thirds of the population are still using alternative traditional medicine in this country [14]. Most commonly practiced CAMs in Bangladesh are herbal, homeopathy, traditional, and religious methods. Sometimes these alternative and traditional methods of treatment reach beyond the boundary of health to the wider environment of society, religion, and culture of the community. Despite the wide acceptance of CAM along with modern medicine, there is still no specific data on the pattern of using these therapies among T2DM patients is available in the context of Bangladesh.

The present study was to determine the rate of CAM use, its associated factors, and the effect on glycemic control among diabetic patients of the northern part of Bangladesh.

## Methods

### Study setting and participants

This cross-sectional study was conducted during November and December 2019 in the outpatient department of Rajshahi Medical College Hospital (RMCH), a tertiary care teaching hospital in the Northern part of Bangladesh, with the well-established outpatient facility. According to hospital records, more than 1000 patients take medical services from the outpatient department of RMCH daily. It is the main treatment facility for the people living in different districts of the northern part of Bangladesh (Rajshahi, Rangpur and Khulna division).

### Sample size determination and sampling

The following formula was used to calculate the sample size for the current study:

$$ n=\frac{z^2 pq}{d^2} $$

where n = number of sample; z = 1.96 for 95% confidence level (CI), p = the proportion of prevalence and d = precision of the prevalence estimate. There was no previous evidence of the prevalence of CAM use among Bangladeshi T2DM patients. However, a Malaysian study reported that the prevalence of CAM use among diabetes patients was 62.5% [8]. A total number of 240 diabetic patients were used as sample for their study and they got the prevalence of CAM use was 62.5%. We did not follow their sample size, we just took the prevalence of CAM for calculating our sample size. We considered *p* = 62.5% = 0.625, so q = 1-*p* = 1–0.625 = 0.375. Taking this into account assuming 10% precision of the prevalence estimate (d = 0.0625) and 95% CI, 231 samples were sufficient for this study. Assuming a 10% nonresponsive rate total of 260 diabetic patients were considered.

Patients were selected using a purposive sampling method. Patients aged more than 18 years and diagnosed as T2DM for at least one year were included in this study. Patients diagnosed as other types of diabetes, diagnosed for less than one year, and the patients with complications who needed hospitalization were excluded. After eliminating incomplete responses data from 244 participants were finally analyzed.

### Data collection instrument and procedure

Data were collected by face-to-face interviews using a structured questionnaire (Additional file: Questionnaire). A written consent was taken from each participant. The questionnaire included patients’ socio-demographic data, diabetes-related information, types of CAM used, and source of motivation for using CAM. The attitudes, beliefs, and perceptions toward CAM were also explored. Results of glycosylated hemoglobin (HbA1c) test from the current visit, when the interview was conducted, were collected from the patients’ medical records. A pilot survey involving 20 patients was conducted to pretest the questionnaire and estimate the likely response rate. The interview was carried out by three trained medical students.

### Outcome variable

The use of CAM was the outcome variable of this study and the sample was classified according to CAM usage such as (i) CAM user (code, 1) and (ii) CAM non-user (code, 0). Another outcome variable in this study was glycemic control; the sample was divided into two classes; (i) poor control (code, 0) and (ii) good control (code, 1). Cut off value of poor glycemic control was set as HbA1c ≥7% and for good glycemic control, it was < 7%. These cut off values were taken from previous evidence of glycemic control among Bangladeshi diabetic patients [15, 16].

### Independent variables

Some socio-demographic and diabetes-related factors were considered as independent variables in this study. Most of the independent variables were selected based on previous studies [7, 8]. The names of variables with group and code are given in Table 1.

**Table 1:** List of independent variables with group and code

Variable	Group	Code	Variable	Group	Code
Age	Below 40 years	1	Residence	Rural	1
	41 to 60 years	2		Urban	2
	60+ years	3	Duration of diabetes	More than 5 years	1
Sex	Male	1		Less than 5 years	2
	Female	2	Complications of diabetes	Yes	1
Religion	Muslim	1		No	2
	Hindu	2	Type of medication	Oral hypoglycemic drug	1
Marital status	Married	1		Insulin	2
	Single	2		Mixed	3
	Widowed/Divorced	3	Frequency of health care center visit	Irregular	1
Education level	Uneducated	1		Regular	2
	Primary	2	Family history of diabetes	Yes	1
	Secondary	3		No	2
	Higher study	4			
Family status (measured by monthly family income (in taka))	Low (<BDT15000)	1			
	Middle (BDT15000–30000)	2			
	Higher (>BDT30000)	3			

### Statistical analysis

Statistical analyses were carried out using SPSS (IBM version 22.0). The qualitative variables were described in terms of frequencies and percentages, and continuous variables in terms of means and standard deviations. The association between socio-demographic or diabetes-related factors and the use of CAM was determined by using Chi-square (χ^2^) test. Multivariate logistic regression was used to identify the most influential predictors of CAM usage. Multivariate logistic regression adjusted for socio-demographic and diabetes-related factors were used to find out the effect of CAM usage on glycemic control.

## Results

### Socio-demographic characteristics

A total of 244 diabetes patients were enrolled in this study. The socio-demographic characteristics and diabetes-related factors are presented in Table 2. The participants were predominantly female with a mean age (SD) of 54.15 (13.18) years. Their mean (SD) duration of diabetes was 8.89 (5.42) years and two-third of them had uncontrolled diabetes. More than 60% of the participants were suffering from diabetes-related complications (Table 2).

**Table 2:** Socio-demographic and disease-related characteristics of the study sample (*n* = 244) and their association with CAM use

Characteristics	Total (n = 244)	CAM users (*n* = 86)	CAM non-users (*n* = 158)	*p*-value
Socio-demographic factors				
Age				
Below 40 years	31 (12.7)	5 (16.1)	26 (83.9)	0.005
41 to 60 years	140 (57.4)	46 (32.9)	94 (67.1)	
60+ years	73 (29.9)	35 (47.9)	38 (52.1)	
Sex				
Male	100 (41.0)	38 (38.0)	62 (62.0)	0.453
Female	144 (59.0)	48 (33.3)	96 (66.7)	
Religion				
Muslim	192 (78.7)	72 (37.5)	120 (62.5)	0.157
Hindu	52 (21.3)	14 (26.9)	38 (73.1)	
Marital status				
Married	184 (75.4)	68 (37.0)	116 (63.0)	0.549
Single	16 (6.6)	4 (25.0)	12 (75.0)	
Widowed/Divorced	44 (18.0)	14 (31.8)	30 (68.2)	
Education level				
None	22 (9.0)	12 (54.5)	10 (45.5)	0.042
Primary	68 (27.9)	28 (41.2)	40 (58.8)	
Secondary	108 (44.3)	34 (31.5)	74 (68.5)	
Higher study	46 (18.9)	12 (26.1)	34 (73.9)	
Family income				
Low	16 (6.6)	12 (75.0)	4 (25.0)	0.001
Middle	130 (53.3)	54 (41.5)	76 (58.5)	
Higher	98 (40.2)	20 (20.4)	78 (79.6)	
Residence				
Rural	108 (44.3)	52 (48.1)	56 (51.9)	0.001
Urban	136 (55.7)	34 (25.0)	102 (75.0)	
Diabetes related factors				
Duration of diabetes				
More than 5 years	84 (34.4)	68 (42.5)	92 (57.5)	0.001
Less than 5 years	160 (65.6)	18 (21.4)	66 (78.6)	
Complications of diabetes				
Yes	148 (60.7)	62 (41.9)	86 (58.1)	0.007
No	96 (39.3)	24 (25.0)	72 (75.0)	
Type of medication				
Oral hypoglycemic drug	121 (49.6)	41 (47.6)	80 (50.7)	0.903
Insulin	69 (28.3)	25 (29.1)	44 (27.8)	
Mixed	54 (22.1)	20 (23.3)	34 (21.5)	
Frequency of health care center visit				
Irregular	110 (45.1)	56 (41.8)	78 (58.2)	
Regular	134 (54.9)	30 (27.3)	80 (72.7)	0.018
Family history of diabetes				
Yes	108 (44.3)	48 (44.4)	60 (55.6)	0.007
No	136 (55.7)	38 (27.9)	98 (72.1)	

### Rate of CAM using patients

It was observed that more than 35% of diabetes patients used CAM for controlling their diabetes. The sex distribution of CAM users was similar, and older patients used CAM more frequently than younger ones. Patients came from rural areas, and living in low-income families used CAM more than their counterparts. Patients diagnosed as diabetic for more than five years, having a family history of diabetes, suffering from diabetes-related complications, and doing irregular follow-up were found to have a higher rate of CAM use. Chi-square test demonstrated that patients’ age group, education level, family income, residence, duration of diabetes, complication of diabetes, frequency of health care visit, and family history of diabetes were the significantly associated factors of CAM usage (Table 2).

Only significantly associated factors provided by the Chi-square test were considered as independent variables in the multivariate logistic regression model. After controlling the effect of other factors, this model demonstrated that uneducated diabetes patients were more likely to use CAM than higher educated patients [AOR = 3.40; 95% CI: 1.170–9.877, *p* < 0.05]. It was noted that patients who lived in low and middle-income families had 8.703 times higher risk for using CAM than higher-income family patients respectively [AOR = 8.703; 95% CI: 2.151–35.216, *p* < 0.01] and 2.041 [AOR = 2.041; 95% CI: 0.998–4.174, p < 0.05]. Patients suffering from diabetes for more than 5 years were more likely to use CAM than their counterparts [AOR = 2.821; 95% CI: 1.338–5.947, p < 0.01]. Rural patients had shown more chance of using CAM than urban patients [AOR = 1.767; 95% CI: 0.903–3.461, *p* = 0.097], and patients who had a family history of diabetes were more likely to use CAM than those who did not have a family history [AOR 1.698; 95% CI: 0.937–3.077, *p* < 0.081] (Table 3).

**Table 3:** Correlates of CAM use in multivariate logistic regression among the study sample

	AOR	95% CI for AOR: Lower-Upper	*p*-value
Age			
Below 40 years	0.543	0.164–1.799	0.317
40 to 60 years	0.650	0.339–1.245	0.194
60+ years	1		
Education level			
None	3.400	1.170–9.877	0.025
Primary	1.983	0.877–4.487	0.100
Secondary	1.302	0.601–2.821	0.504
Higher study	1		
Family income			
Low	8.703	2.151–35.216	0.002
Middle	2.041	0.998–4.174	0.045
Higher	1		
Residence			
Rural	1.767	0.903–3.461	0.097
Urban	1		
Duration of diabetes			
More than 5 years	2.821	1.338–5.947	0.006
Less than 5 years	1		
Complications of diabetes			
Yes	1.189	0.613–2.307	0.609
No	1		
Health care center visit			
Irregular	1.210	0.651–2.249	0.547
Regular	1		
Family history of diabetes			
Yes	1.698	0.937–3.077	0.081
No	1		

### CAM-related characteristics among CAM users

The highest number of CAM users (67.4%) used herbal products (such as *Gynura procumbens*, locally known as ‘diabetes tree’, fenugreek, bitter gourd, etc.), followed by homeopathic medicine (37.2%) and traditional/ religious methods (9.3%) (Table 4).

**Table 4:** CAM related characteristics among CAM users (n = 86)

CAM related characteristics	Number	Percent
Type of used CAM		
Herbal products	58	67.4
Leaf of *Gynura procumbens*	42	48.8
Fenugreek	23	26.7
Bitter gourd	17	19.8
Turmeric	14	16.3
Okra	26	30.2
Others	9	10.5
Homeopathic medicine	32	37.2
Traditional/religious method	8	9.3
Multivitamin/food supplements (other than prescription)	5	5.8
Others	3	3.5
Source of information about CAM		
Friends/ neighbors	44	51.2
Family members	18	20.9
Media/ advertisements	10	11.6
CAM practitioners (Kabiraj/Herbal/ Homeo practitioner)	12	13.9
Diabetes doctor	2	2.3
Mode of CAM use		
As alternative therapy	24	27.9
As complementary therapy	62	72.1
Reasons of CAM use		
CAM helps in diabetes control	38	44.2
Easily available and cheap	24	27.9
Dissatisfied with Conventional medicine	8	9.3
Less side effects	6	7
No specific cause	20	23.3
Self-reported efficacy of CAM		
Very good	12	14
Good	28	32.6
Nothing significant	46	53.5
Experienced any side effect		
No	74	86
Yes	12	14
Reported side effects (*n* = 12)		
Abdominal discomfort	6	50
Nausea, vomiting	4	33.3
Vertigo	2	16.6
Hypoglycemia	2	16.6
Others	3	36

Most of the CAM users (72%) reported that they got information about CAM from their friends, neighbors, and family members, while others reported CAM practitioners (such as ‘kabiraj’ or traditional healers, herbal or homeopathic medicine practitioners) and mass media or advertisement of CAM products (Table 4).

Table 4 also describes the characteristics of using CAM among study participants. Almost 28% of CAM users reported that they used CAM as an alternative to mainstream medicine while others reported that they used CAM on a complementary basis. CAM users believed that CAM would help in better diabetes control and easy availability and lower cost were the most commonly cited reasons for using CAM (44.2 and 27.9% respectively) followed by dissatisfaction with conventional medicine (9.3%) and fewer side effects (7%). More than 23% of users used CAM without any specific reason. Almost half of the users reported the efficacy of CAM as ‘nothing significant’, while others reported as somewhat good, 14% of CAM users experienced side-effects. Gastrointestinal upset, vertigo, and hypoglycemia were commonly reported side-effects of CAM (Table 4).

### Effect of using CAM on glycemic control

Almost 76% of CAM users had poor glycemic control, while the rate was 61.4% among CAM non-users. It was found that a greater number of non-CAM users (38.6%) could control their diabetes better than CAM-users (24%). The simple logistic regression model showed that non-CAM users had 1.946 times better control of their diabetes than CAM users. When we controlled the effect of socio-economic, demographic, and diabetes-related factors, it was observed that non-CAM users were more likely to control their diabetes than CAM user patients [AOR = 2.255; 95% CI: 1.147–4.437, *p*-value < 0.05] (Table 5).

**Table 5:** Effect of using CAM on glycemic control among study sample (n = 244)

Glycemic control	Poor	Good	COR (95% CI)	p-value	AOR^a^ (95% CI)	*p*-value
CAM non-users	97 (61.4)	61 (38.6)	1.946 (1.082–3.501)	0.033	2.255 (1.147–4.437)	0.018
CAM users	65 (75.6)	21 (24.4)	1		1	

^a^adjusted for socio-demographic factors, duration of diabetes and frequency of health care center visit

## Discussion

The present study provided insight into the prevalence and sociodemographic and diabetes-related factors of using CAM among T2DM patients of the northern part of Bangladesh as well as the patients’ attitude towards using these. The overall prevalence of using CAM found in our study was 35.2%. Being a localized study in a hospital serving patients mainly from the northern part of Bangladesh (Rajshahi, Rangpur, and part of Khulna division), findings of this study may not be inferential for all the patients of different regional and cultural background. Despite the fact, it was the first report on the prevalence of using CAM among Bangladeshi diabetic patients according to our knowledge. However, the rate was much lower than the estimated rate of CAM use among the general population of the country, which was 70 to 75% in a previous study [14]. The finding was also lower compared to the different countries of Asia and North America. For example, prevalence of using CAM among diabetic patients in neighboring India was 67.7% [17], in Malaysia 62.5% [8], in Taiwan 61% [18], in Korea 65% [19], in Thailand 47% [20] and in Turkey 41% [21]. Among American countries, in the USA the prevalence was 57% [22] and in Mexico, it was 62% [23]. The prevalence of using CAM among our study populations was higher compared to the UK (17%) [24] and Australia (23.6%) [25]. Different socio-cultural orientations, patients’ health beliefs, and attitudes, as well as the health care system and access to modern medicine, could be attributable to the regional variation of using CAM. Moreover, variations in defining CAM in different study designs might have also contributed to this variation.

Patients from lower-income families, without education and suffering from diabetes for a longer time, were more likely to use CAM. Longer duration of disease was also reported as a predictor of using CAM in the USA and India along with older age and higher educational attainment and higher family income [17, 26], though our finding was opposite in case of educational and socioeconomic status. Different studies from Asian countries did not find a significant association of using CAM with socio-demographic or disease-related characteristics [7, 8].

Herbal products were reported as the most commonly used CAM followed by homeopathic medicine. Herbal products were also reported as the most popular alternative therapy in different Asian countries [7, 8, 17]. The rich floral diversity of this region has constituted indigenous herbal medicine as an important component of the primary healthcare system of Bangladesh [27]. Homeopathic medicine is also frequently used in this county. Lower cost and patients’ positive perception about this therapy as effective for long term cure and fewer side effects have made this treatment widely acceptable [28]. However, traditional and religious methods, acupuncture, energy therapies like reiki, and mind-body interventions like yoga or tai-chi were less commonly practiced in this country compared to other Asian countries like Malaysia and Taiwan [8, 18]. Family and friends were the most frequently reported sources of information about CAM, which was consistent with the findings of other Asian countries [7, 18]. Most of the patients attending the present study used these alternative therapies as complementary to conventional treatment. A similar finding was reported in a study from Lebanon [7]. This is an important concern for the physicians to look for whether the patients are using other alternative therapy, as it can be a potential source of drug interaction [11] as well as patients’ non-compliance to the therapy and ultimately hamper the optimum glycemic management.

Positive attitude toward the efficacy of CAM and easy availability and low cost were commonly reported reasons for using CAM. It is very practical to look for alternative and cheap therapy in a lower-middle-income country like Bangladesh, especially among the patients of a government hospital, where most of the patients belong to lower socio-economic group, and diabetes management causes a high out-of-pocket expenditure [29, 30]. Surprisingly, only a few patients were dissatisfied with conventional treatment and more than half of the patients who were using CAM reported that these agents were not effective in diabetes control. Moreover, almost 14% of patients who were using CAM reported different types of side effects, most commonly gastrointestinal discomfort. These findings were similar to the previous reports from Malaysia [7], Thailand [8], and Lebanon [17]. Perhaps most of the users were using these products as an experiment for self-care of diabetes as reported by a study from Lebanon [7]. Another study from the USA reported similar findings and hypothesized that patients were using alternative therapies influenced by their cultural values, beliefs, and philosophical orientations toward health and life rather than being dissatisfied with conventional medicine [31]. Moreover, as conventional management of diabetes needs a disciplined lifestyle for diet, lifestyle, and behavior, which is often hard to maintain, these patients try to compensate by using CAM as they believe it can offer more personal autonomy and control over their disease [32].

According to the finding of our study, using CAM had a negative impact on glycemic control. This issue remained ambiguous as studies reported the different effects of CAM on diabetic management. Several studies conducted among diabetes patients reported that using CAM was associated with positive health behavior and better self-management of diabetes, as it may be part of a broader lifestyle [6, 11, 31]. In these studies, CAM use was associated with higher educational attainment that could be an independent predictor of good glycemic control, whereas, in our study, patients with lower educational attainment mostly used CAM. Moreover, these studies did not report any quantitative evidence on glycemic control. However, there are some evidences from different countries like Malaysia and Israel, reporting that consumers of CAM had no significant difference of HbA1c compared to non-users, though these studies compared only the mean of HbA1c, which was not adjusted for other potential variables [8, 33, 34].

### Strength of the study

The present study was one of the very first attempts to determine the rate and influencing factors of using CAM among the diabetes patients of Bangladesh. This provides important evidence on the effect of CAM on glycemic control of diabetes patients that can guide the clinicians for optimum diabetes management. The population included in this study represents a great portion of the overall diabetes patients of Bangladesh, so the result can be inferred for the majority of the patients.

### Limitation of the study

A number of limitations of this study are worth mentioning. This study was conducted in a tertiary care government hospital of northern Bangladesh that may not reflect the original picture of the total diabetic population of the country. Another important limitation was its sample size was relatively small for robust statistical inference. The predicted prevalence of CAM usage from different evidences that was used to calculate sample size was much higher than the actual prevalence found in this study. A greater sample size would be considered for better statistical power, which was not possible for our limited resources. The prevalence and patterns of CAM use among patients who are not on regular follow-up by physicians could not be figured out. Although participants were assured of the confidentiality of their responses it could not be ascertained that patients did not experience the social desirability bias, potentially altering their answers to satisfy their health care providers. A more extensive study including different socio-cultural factors and both quantitative and qualitative method is necessary for a better understanding of the patients’ perspectives of using CAM. The definition of good glycemic control used here (HbA1c < 7%) was previously used and proved sensitive for the diabetic patients of Bangladesh [15, 16]. However, the change of HbA1c might be a better index for defining glycemic control, but due to the cross sectional design of the study, follow up of the glycemic control was not possible, which is an important shortcoming of the study. Further studies should consider the follow-up of the glycemic index.

## Conclusions

From the perspective of increasing interest in CAM among diabetic patients, our study provides an insight on the prevalence and pattern of using different CAM among the diabetes patients of Bangladesh as well as shows its negative impact on glycemic control. Awareness about the ineffectiveness and side-effects of irrational use of CAM should be raised among diabetic patients by educational programs and health education measures.

## Supplementary information

**Additional file 1.** Questionnaire.

## Data Availability

Patient-level data will be available on request, provided that approval is given from the Ethical Review Committee of Rajshahi Medical College, Rajshahi, Bangladesh.

## Abbreviations

CAM: Complementary and alternative medicines
RMCH: Rajshahi Medical College Hospital
COR: Crude odd’s ratio
AOR: Adjusted odd’s ratio
CI: Confidence interval
