# Supplementary material for: A hospital-based study on complementary and alternative medicine use among diabetes patients in Rajshahi, Bangladesh
Source: BMC Complement Med Ther. 2020 Jul 13;20:219. doi: 10.1186/s12906-020-03021-3 (PMC7359228; doi:10.1186/s12906-020-03021-3)
Supplement: Supplementary file 1 — Additional file 1. Questionnaire. [file 12906_2020_3021_MOESM1_ESM.docx]

**Questionnaire**

**Demographic data**

1. ID no.:________________
2. Name:___________________________
3. Age: ________ years
4. Sex: € Male € Female
5. Religion: ________________
6. Marital status:

- Single
- Married
- Divorced/Widowed

1. Education:

- None
- Primary
- Secondary/ Higher secondary
- University graduate

1. Occupation:
2. Socioeconomic status:

- Lower income (<BDT10000)
- Middle income (BDT10000-30000)
- Higher income (>BDT30000)

1. Residence: € Rural € Urban

**Diabetes data**

1. Duration of DM: __________years
2. Lab report

| HbA1c |  | % |
| --- | --- | --- |
| RBS |  | mmol/L |
| FBS |  | mmol/L |
| PPG |  | mmol/L |
| S. creatinine |  | mg/dl |

1. Complications

- Neuropathy
- Nephropathy
- Retinopathy
- Macro vascular complications
- No complication

1. Type of medication

- Oral
- Insulin
- Both

1. Frequency of visiting doctor

- Regular (at least once within 6 months)
- Irregular

1. Family history of DM: € Yes € No

**CAM related data**

1. Do you use any type of CAM (e.g. herbal products or homeopathy) for your diabetes?

€ Yes € No

1. What is the mode of use?

€ Complementary € Alternative

1. Type of CAM

- Herbal (specify)___________________
- Homeopathic medicine
- Food supplements & multivitamins (other than prescription)
- Traditional/ religious methods
- Others (specify)___________________

1. Who suggested you to use CAM or from where you have got information about CAM?

- Family members
- Friends/ neighbors
- Media/ advertisements
- Diabetes doctor
- CAM practitioners (Kabiraj/Herbal/ Homeo practitioner)

1. Why do you use CAM?

- CAM helps in diabetes control
- Less side effects
- Easily available and cheap
- Dissatisfied with Conventional medicine
- No specific cause
- Others __________________________

1. Self-reported efficacy of CAM

- Very good
- Good
- Nothing significant
- Worsening condition

1. Have you ever faced any side effect of CAM?

€ Yes (Specify_____________________)

€ No

Thank you very much for providing your information.

Data Collector’s Signature Responder’s Signature
